# An Australian model of the First 1000 Days: an Indigenous-led process to turn an international initiative into an early-life strategy benefiting indigenous families

**DOI:** 10.1017/gheg.2016.7

**Published:** 2016-06-27

**Authors:** R. Ritte, S. Panozzo, L. Johnston, J. Agerholm, S. E. Kvernmo, K. Rowley, K. Arabena

**Affiliations:** 1 Onemda VicHealth Koori Health Group, Centre for Health Equity, Melbourne School of Population and Global Health, The University of Melbourne, Melbourne, Australia; 2 Indigenous Health Equity Unit, Centre for Health Equity, Melbourne School of Population and Global Health, The University of Melbourne, Melbourne, Australia; 3 Research, Innovation and Commercialisation, The University of Melbourne, Melbourne, Australia; 4 Institute of Clinical Medicine, Faculty of Health Sciences, The University of Tromsø: The Arctic University of Norway, Tromsø, Norway

**Keywords:** Aboriginal and Torres Strait Islander health, families, Indigenous methodologies, maternal and child health

## Abstract

Internationally, the 1000 days movement calls for action and investment in improving nutrition for the period from a child's conception to their second birthday, thereby providing an organising framework for early-life interventions. To ensure Australian Indigenous families benefit from this 1000 days framework, an Indigenous-led year-long engagement process was undertaken linking early-life researchers, research institutions, policy-makers, professional associations and human rights activists with Australian Indigenous organisations and families. The resultant model, First 1000 Days Australia, broadened the international concept beyond improving nutrition. The First 1000 Days Australia model was built by adhering to Indigenous methodologies, a recognition of the centrality of culture that reinforces and strengthens families, and uses a holistic view of health and wellbeing. The First 1000 Days Australia was developed under the auspice of Indigenous people's leadership using a collective impact framework. As such, the model emphasises Indigenous leadership, mutual trust and solidarity to achieve early-life equity.

## Introduction

The period from conception through to a child's second birthday – the first 1000 days – is an ideal time in which to shape a healthier future for that child [1]. The internationally recognised 1000 days movement [1] was set up to improve maternal and infant nutrition during this time in a child's development. By focusing on reducing malnutrition in mothers and children, the 1000 days movement has combined evidence-based medical care and social support to families and children experiencing vulnerability due to economic, psychological and social inequalities. Strategies emanating from this approach have now been implemented in the USA [1] and in countries throughout Asia [2], Europe [3], South America and Africa [4] Interventions are having demonstrable outcomes [1], such as significantly reducing the human and economic burden of communicable diseases and the long-term risk of developing some non-communicable and chronic diseases, and improving educational achievement, earning potential and a nation's gross domestic product.

Poor nutrition during early life is well recognised as having the potential to cause irreversible damage to a child's neurological, immune and physical development [5]. However, improved nutrition alone will not address the current poor health and wellbeing status of Indigenous children in Australia and elsewhere – a broader, holistic and cultural perspective is needed [6]. This focus on the first 1000 days is particularly important among Australia's Indigenous population because of the increasing number of children from Indigenous families experiencing periods of vulnerability [7], exposure to which has far-reaching developmental, health and economic outcomes over the life-course.

Many early intervention supports, however, are not always available or accessible to babies and families during these times of vulnerability [8]. Despite almost a decade of Australia's ‘Close the Gap’ campaign, aimed at addressing Indigenous’ disadvantage [9], there are mixed opinions as to its effectiveness [10–12]. Far too many Indigenous children are still living in complex family situations, or at heightened risk in households experiencing entrenched disadvantage and seasonal variations in their capacity to live in sustainable, just and healthy families and communities. This is largely because of un(der)employment [13], the effects of climate change [14], the repercussions of trauma [15] and other problems such as substance misuse, mental illness [16], disabilities [17] or family violence [18].

Australia's only Aboriginal Children's Commissioner recently reviewed the cases of a 1000 Indigenous children in out-of-home care in the Australian state of Victoria, and found that parental drug and alcohol use and male-perpetrated family violence contributed to their removal from families into institutional care [19]. Among children under 12 months of age, the Commission found that children born to families during periods of heightened risk were subject to poorer health and cognitive development for reasons that cannot solely be attributed to under-nutrition [20]. Thus, a nutritional intervention alone would be an inadequate response to these children's situation: a broader articulation of the 1000 Days movement is required in the Australian context to ensure life-long good health and wellbeing [21–25].

The family life of Indigenous people is predominantly centred around complex kinship systems and clan structures, with clear lines of rights and obligations to others. Until recently the education and socialisation of young Indigenous children took place within the rhythms of family life, extended family and Country [26]. Indigenous communities intrinsically value children and these values are represented by agencies that are strong advocates for the protections and recognition of the rights of children [27–33]. However, these values have been radically disrupted for some families, particularly those who suffered from policies that resulted in the separation of children from their families, the destruction of extended family networks and decades of living in oppressive circumstances – as evidenced by poor health and early deaths, sub-standard housing, poor educational outcomes, high unemployment and large numbers of Indigenous people in custody [34–37]. Despite these hardships, families remain the primary and preferred site for developing and protecting culture and identity for Indigenous children and for achieving good health and wellbeing outcomes for future generations [28]. This view has been reinforced by national Indigenous health leadership stating that culture, community control, family empowerment and Indigenous-led solutions from inside the community are those that will make the difference [38].

This paper describes the engagement process undertaken at a national level to collect and consolidate the evidence needed to build on the ideas behind the 1000 days movement and develop a model for the Australian context. A continuous process of national and regional engagement activities brought together Indigenous families, communities and organisations with other key stakeholders to discuss what an Australian interpretation of the first 1000 days might look like. This engagement process was premised on the belief that no single policy, government department, organisation or program can solve the increasingly complex social issues faced by Indigenous families. It has facilitated a new approach, which calls for multiple organisations from different sectors to come together around a common agenda, and align their efforts under leadership arrangements that privilege the voices and strategies adopted by Indigenous people.

## Methods

Led by Indigenous scholars at the University of Melbourne the engagement process employed Indigenous methodologies that centralised culture and wellbeing to the health Indigenous peoples, and guided the development of a vision and strategy for our own model – First 1000 Days Australia [39–43]. The process was akin to the collective impact framework [44] to achieve large-scale progress against urgent and complex problems of our time. This approach was adopted because of the need for radical transformation in, rather than incremental change to, the health and wellbeing of Indigenous children and their carers [26].

Over the course of 2015, four formal national symposia – Scientific Symposium, Researchers’ Forum, Community Governance Symposium and Policy and Implementers’ Symposium – were held to investigate the inherent possibilities in applying the 1000 days international model to resolve issues faced by Australian Indigenous families. Organisers invited and linked key stakeholders and organisations, which included Indigenous families, Elders and representative organisations, with scientific researchers from universities and other peak research institutes, front-line workers (such as early learning educators, social workers, midwives and community workers), policy makers from local, State and Federal governments, health economists and representatives from non-government organisations. The Symposia, details of which have been reported [45–48], also attracted representatives from Indigenous communities in Indonesia and Norway.

Symposia participants listened to and discussed the latest research involving the health and wellbeing of Indigenous children and families [45–48]. Presentations included current research and programs that address the impact of out-of-home-care [20] maternal nutrition [25] the neuroscience of infants [49] the long-term impacts of early childhood experiences [50] and epigenetics; the capacity of infants to begin structured learning earlier than previously supposed [51]; building the capabilities of adult caregivers in vulnerable families [52] particularly in adolescence [53]; developing executive function and self-regulation skills in children [54]; and building cultural security [55]. Participants workshopped, in small directed groups, what an Australian model of the First 1000 Days would look like, the potential areas in which to develop strategies and how the outcomes and impacts of the model could be measured.

Each successive symposium built on the previous one in an iterative process. Researchers were guided by the scientific terms of reference developed as a result of the initial Scientific Symposium, and came up with several research themes at the subsequent Researchers’ Forum. These were then discussed, unpacked and amended by community representatives at the Community Governance Symposium. Following this, policy makers were asked to work through the amended research themes and how this research could best be implemented into relevant policy that acknowledged the dynamics and diversity of Australian Indigenous communities [56]. At the conclusion of each of the symposia, recordings of the presentations were edited and notes from the group work thematically summarised into a report, with key considerations for the development a First 1000 Days Australia model [45–47, 56].

## Results

A total of 323 participants, representing 107 organisations attended the four symposia over the course of the year (see Table 1 for details of attendees). Thirty per cent of these organisations were Indigenous, as were almost 40% (38.7%) of the participants. Overall, participants recognised that to have a positive impact on the future prosperity Indigenous peoples, a First 1000 Days Australia model had to broaden the original framework of nutrition and maternal health to include a holistic and ecological approach [57, 58]. Participants highlighted the importance of family-strengthening initiatives, the crucial role played by men in raising children, antenatal and early years’ engagement, building the capacity of both families and the health workforce and generating empirical evidence for the future wellbeing of the coming generations. Further, they recognised the lack of attention currently given to the period from pre-conception to birth and extended the first 1000 days of life period to include pre-conception.

**Table 1.** Number of symposia participants^a^ representing 107^b^ different organisations and/or communities

	Scientific		Researchers		Community Governance		Policy and implementers	
Organisation	Frequency	Proportion	Frequency	Proportion	Frequency	Proportion	Frequency	Proportion
Indigenous led Services and service support	8	8.2%	10	15.0%	16	23.9%	10	10.2%
Services (health, early childhood)	2	2.0%	0		7	10.4%	10	10.2%
Research institutions (Indigenous specific)	22	22.4%	22	36.7%	19	28.4%	18	18.4%
Research institutions (non-Indigenous specific)	42	42.9%	0		8	11.9%	18	18.4%
Health institutions	2	2.0%	2	3.3%	6	9.0%	7	7.1%
Industry	4	4.1%	22	36.7%	1	1.5%	0	–
NGO/NFP	4	4.1%	0		3	4.5%	9	9.2%
Local government	0	–	0		1	1.5%	6	6.1%
State government	13	13.3%	0		4	6.0%	13	13.3%
Commonwealth government	0	–	3	5.0%	0	–	1	1.0%
Other(such as media)	1	1.0%	1	1.7%	2	3.0%	6	6.1%
Total	98	100.0%	60	100.0%	67	100.0%	98	100.0%

a Participants across the four symposia who attended more than one symposium are counted separately (*N* = 323).

b Organisations across the four symposia are represented only once (*N* = 107).

In addition to a collective agreement for a focus on comprehensive primary health care [57, 58] maternal and child nutrition and early-life literacy, the symposia raised five interconnected themes. Community governance – to ensure direction, leadership and decision making remained under Indigenous control – was seen to be first and foremost an essential requirement of any research to be done or strategies to be implemented in Indigenous communities. It was collectively determined that strategies under the auspice of First 1000 Days Australia needed to address the family environment, increasing antenatal and early years’ engagement and service use and provision. Finally, evidence was also required to inform policy and to show empirically the health benefits of taking the holistic approach advocated by First 1000 Days Australia so as to balance the current biomedical approaches used to build evidence in health research [59].

### Community governance

The community governance discussion highlighted that any First 1000 Days Australia strategies should be led by Indigenous people as co-designers, co-implementers and co-knowledge translators of research and outcomes at national and regional levels. Such community governance would need to be informed by scientific evidence to ensure that decision making is guided by best practice [60] and up-to-date research. The establishment of a national interdisciplinary Community Governance Committee was recommended to oversee and ensure that all programs to be developed, implemented and/or translated as part of First 1000 Days Australia are culturally appropriate, safe and embed cultural protective factors for mothers, fathers and children through a strong connection to culture, Country, community, and family members and other key people [60]. This committee would comprise members of Indigenous organisations, community representatives and Elders, and policymakers. Also vital to ensuring good governance would be transparent appointment and decision-making processes for the Community Governance Committee members with open communication between researchers and the regional sites implementing First 1000 Days Australia strategies.

Participants discussed that a local governance process would have to be in place to determine the exact nature of appropriate, community-specific interventions and capacity-building strategies tailored to the requirements of the region and its respective community. This would mean, ultimately, that families participating in First 1000 Days Australia programs would decide which local strategies and interventions would be the most suitable for them. Participants highlighted that, with strong community governance embedded in regions implementing First 1000 Days Australia strategies, the model had potential to increase both the opportunities for community leadership in agenda setting and decision making, and the cultural responsiveness and capacity of service systems to meet the needs, and recognise the diversity and heterogeneity, of Indigenous peoples and communities across Australia.

### The family environment

When discussing the family environment, participants grounded the First 1000 Days Australia model on the premise that the role of protecting children is best undertaken by the family – a multigenerational, non-biological and traditional model of family that includes mothers, fathers and/or care givers, grandparents and other relatives. In building strong, resilient families, symposia participants emphasised the need to include strengths-based approaches and cultural measures of wellbeing [61]. Community leadership in this area is, therefore, required to shift from a dependence on *child and maternal health* services to *maximising protective factors in families*. Participants recommended that effective supports for families of Indigenous children during the first 1000 days period are those that enhance relationships between these children and their parents/care givers by taking a case management approach prior to conception to the age of 2 years.

### Increasing antenatal and early years’ engagement

Current models of innovative antenatal engagement with Indigenous communities include incentivised programs and home visits for mothers and infants [62, 63]. However, symposia participants highlighted the additional need for further engagement with men during their transition to becoming fathers. Participants understood the importance of antenatal engagement between Indigenous families and health service providers, and advocated for a whole-of-service of approach that included counselling, early learning, education, correctional services, housing, drug and alcohol services, and family empowerment programs. They also nominated alternative approaches to health and wellbeing other than those delivered through service agencies, such as accessing micro-business solutions, family-based and local enterprises, personal coaching and family mentoring.

### Service use and provision

The service use and provision theme identified at the symposia focused on building capacity with parents, families and the workforce during the first 1000 days that not only recognised the heterogeneity between urban, regional/rural and remote locations, but also the diversity among nations, language groups, expressions of culture, experience of native title and connection to Country, as well as the individual experiences of families enrolled in the First 1000 Days Australia programs. Participants also asked that consideration be given to the development of a First 1000 Days Australia workforce, the provision of targeted education both to engage and to support local and regional implementation of programs, and the building of a national network of First 1000 Days Australia practitioners. Furthermore, they recommended service providers explore alternative approaches that would lead to a broadening by services away from a clinical service provision focus to one that facilitates improved access to services guided by the principles of comprehensive primary health care using a case management approach centred on family empowerment programs.

### Data for evidence

Symposia participants called for a scientifically robust and decolonised evidence base to be built as a legacy of First 1000 Days Australia, thereby ensuring that any impacts from the strategies implemented were measured appropriately [64, 65]. They stressed the need for this legacy to be led by Indigenous researchers and to be built upon a systematised approach to generating, collecting, linking and using data. This would include the development of appropriate and rigorous measures for cognitive, growth and behavioural development, education, health and cultural wellbeing of Indigenous children and families.

Participants also pushed for the development of an Indigenous-led and governed, pre-conception longitudinal study, using a multi-generational Indigenous definition of family, to investigate the impacts of strategies under the auspice of First 1000 Days Australia. Further, they called for an extension of a traditional epidemiological observational study to include an intervention element rather than just observing and reporting on the outcomes associated with families experiencing vulnerability over time. Participants identified that data generated from First 1000 Days Australia programs would catalyse improvements in policy, practice, family empowerment, business and whole-of-government services that will enable Indigenous children, families and communities to flourish. However, they also recognised that to facilitate such a study, a large investment is required to improve the coordination, collection and access of population data, as well as working with governments and the Indigenous sector to ensure the acceptability of the data collection methods.

## Discussion

The year-long process of this Indigenous-led campaign of engagement [45–48] resulted in a broadening of the international 1000 days movement to create an Australian model with a holistic framework that supports resilience within, and is appropriate for, Indigenous communities. The First 1000 Days Australia model was built by adhering to Indigenous methodologies [43, 66, 67] a recognition of the centrality of culture that reinforces and strengthens families [38], and a holistic view of health and wellbeing [57, 58]; This dynamic process has ensured that the First 1000 Days Australia is based on strengths-based empowerment and not deficit [68]. Specifically, the First 1000 Days Australia model includes strong community governance at regional and national levels, thereby obligating researcher accountability and binding participating organisations to a shared vision and set of strategies focusing on the family environment; increased antenatal and early years’ engagement; and service use and provision. Further, the engagement process has enabled the initiation of an evidence base that embeds culture and an Indigenous perspective on health and wellbeing into a Longitudinal Study that starts in families prior to conception. The process of engagement and resultant Indigenous-led First 1000 Days Australia model has the potential to be a benchmark for all people experiencing vulnerability and disadvantage, because of the place-based process to inform and engage Indigenous peoples internationally.

The international concept of the 1000 days has been broadened from a focus on maternal and infant nutrition to a holistic health and wellbeing framework more appropriate for Indigenous communities. This broadened concept was established by ensuring the centrality of culture and a holistic view of health and wellbeing that drove the development of an Indigenous methodological framework. The process combined scholarly, community and organisational engagement [69] and was premised on Indigenous cultural leadership, Elder wisdom and authority, diversity, inclusiveness, narrative practices and a valuing of family-centred approaches, partnerships and collaborations [40, 70, 71]. Included in this process was a recognition of Indigenous people's holistic concept of health [57], which is not just the physical wellbeing of an individual but also the social, emotional and cultural wellbeing of the whole community so that each individual is able to achieve their full potential [72]. In addition, a key focus of the methodological framework ensuring human rights through the implementation of the key principles in the United Nations Declaration on the Rights of Indigenous Peoples [73], which governs the rights of children [27].

Indigenous methods of engagement during the symposia included narrative practices in which multiple views and the multiple voices (yarning with purpose) of participants’ were heard [19, 40]. This was a core component of a decolonisation [74] process that did not privilege particular voice, but instead asked participants to operate as a collective in which there were no hierarchies nor status. Everyone was held as equal, and equally accountable for the quality of their contribution. This Indigenous strategy is more concerned with the intersections [75] between different knowledges and how the synthesis of these knowledges can contribute to solutions [76] for Indigenous families. For example, early onset vascular dementia [50] was discussed – with representatives from the perspective of health, law, community justice, Elders, drug and alcohol misuse, literacy, adolescent health specialists and gerontologists and everyone given the opportunity to learn from each other and propose a collective solution on that was supported across all these viewpoints. Elder engagement throughout the development of the Australian model provided an investment in principled leadership [77], whereby moral and ethical dimensions were considered and the language of deficit were reframed to those of strength. The final element of the Indigenous methodology was to invest in collaborative rather than competitive processes [44]. Underpinning this is the notion of collective action [44] through an intersection of disciplines, cultures, generations, life experiences and capacities to make good on the promise of health equity for all.

The collective impact framework [44] facilitated a new approach to capacity building, family empowerment, systems reform, population-level strategies interventions and the promotion of robust evaluation. The key elements of the engagement process created a common agenda for change including a shared understanding of the problem and a joint approach to solutions and the development of data collection and results across all the participant groups; the conduct of mutually reinforcing activities, open and continuous communication to create common motivation and the provision of staff to service the entire initiative and coordinate participating organisations and agencies. As a result, there was an expansion of the international 1000 days movement that encompassed maternal and infant nutrition within a holistic view of health and wellbeing prior to conception. The Australian Model of the First 1000 Days places nutrition within the family context and aims to provide a coordinated, comprehensive intervention to address the needs of Indigenous children from pre-conception to 2 years of age.

Specifically, the expanded concept of the 1000 days movement includes strong community governance at regional and national levels, thereby obligating researcher accountability and binding participating organisations to a shared vision and set of strategies focusing on the family environment, increasing antenatal and early year's engagement, and service use and provision that addresses preconception, conception and pregnancy, nutrition, resilience, parenting, health literacy, and drug and alcohol issues. The Model moves beyond a service or programmatic responses to include economic approaches, such as family enterprises and micro business solutions for families, in a bid to generate alternative sources of income. Partners are able to articulate their role in offering services guided by the principles of comprehensive primary health care [57, 58] and their collaboration with other partners to establish shared measurement practices, build public will, advance policy and mobilise funding. The Community Governance strategy means that the ownership, control and decision making are made together by both Indigenous academics and researchers and the participating local community members.

To show the impacts of the First 1000 Days Australia on Indigenous families, an evidence base that embeds culture and an Indigenous perspective on health and wellbeing is being established in the form of an Indigenous led Longitudinal Study starting in families during the pre-conception. As far as we are aware, the proposed longitudinal study will be the first Australian Indigenous led cohort study to address and describe the vulnerabilities as well as the protective factors of Indigenous families across multiple settings [22, 78–80] that will equally value the biomedical paradigm of health research and Indigenous knowledges and methodologies [39, 59]. Likely indicators will include measures of wellbeing (such as identity, culture, community, individual and family) and clinical and biological indicators of growth, stress and early markers of disease, but further details and success-based outcomes are currently being developed with further engagement with Indigenous stakeholders and academic partners. In addition, data from administrative data sets and other cohorts will be linked to find broader indicators of success on parental outcomes and school outcomes such as the National Assessment Program Literacy and Numeracy results [81].

Indigenous populations globally share common experiences of colonisation and profound transitions in lifestyle and health [82], but also have similarities in social and cultural patterns such as child-rearing practices, kinship systems, a closeness to nature and belief systems. Although the social transformation wrought by colonisation has mainly led to disadvantaged living conditions and a high burden of disease for Indigenous groups, the impact of this varies [18, 82, 83]. Some Indigenous peoples experience no or few health disparities compared with their non-Indigenous counterparts, and in fact have excellent health [83]. Few, if any, international child studies have investigated Indigenous child health and development across Indigenous groups, with a particular focus on the social and cultural determinants that promote healthy development in children [18]. Even more importantly, there have been no studies on the development and implementation of strategies under the auspice of First 1000 Days Australia culturally adapted to Indigenous children in general. First 1000 Days Australia is the only program in this area to focus on Indigenous children across nations, as the Indigenous knowledges and methodologies underpinning the model's engagement process is replicable and relevant to Indigenous peoples across the world.

A major strength of the process of engagement to determine the Australian model of the First 1000 Days is that it has been led by Indigenous scholars in partnership with Indigenous organisations, and has been constructed using Indigenous methodologies. The engagement process has led to a regionally-based approach scaled from a household level with range of strategies, which are supported through data linkage, health service system reform, co-ordination and integration of early life focussed services. These place-based strategies are further supported by nationally responsive policies and have become a focal point for national collaborations.

A further strength of the engagement process to determine the Australian Model of the First 1000 Days is that it has been led by Indigenous scholars in partnership with Indigenous organisations, and has been constructed using Indigenous methodologies. The benefit of this process is that it has created a level of trust with organisations wishing to be a part of the First 1000 Days Model prior to its research implementation phase. The benefit of this process is that it has created a level of trust with organisations wishing to be a part of First 1000 Days Australia prior to its research implementation phase. However, because the model is so overarching and comprehensive, it runs the risk of selective elements being cherry picked out of the model and thus unable to fulfil its promise of comprehensive primary health care [84] Another weakness is reflected in the current political categorisations of health care and role determination. The Australian model may be too ‘un-siloed’ for one government department to take a political lead and, therefore, it may not be a sustainable process.

First 1000 Days Australia is a nation-building exercise in which strategies under its auspice are based within the family environment, address social and cultural issues, build capacity in families, integrates services and creates a First 1000 Days Australia workforce. The Australian model's innovation lies in its comprehensive and holistic approach to addressing the underlying social determinants of health and the context of health outcomes embedded within Indigenous families. Also vital is its ability to facilitate collective impact on issues affecting the health and wellbeing of Indigenous families by incorporating an international nutrition initiative within a context of thriving and engaged families and community wellbeing. Enabling an evidence base to grow from a cultural and Indigenous led paradigm that uses and values Indigenous knowledges and engagement processes, along with evidence-based health care that includes biomedical research relating to practice in health care [59], has implications not just for Indigenous peoples, but for all families experiencing vulnerabilities globally. The promise of health equity and resilient families is dependent on a broader articulation of early disease prevention, and of families being the locus of nation building and the key implementers of health gains for the next generation.

## References

[1] 1000 Days. Why 1000 Days? (http://www.thousanddays.org/about/). Accessed 12 July 2015.

[2] Kattula D, The first 1000 days of life: prenatal and postnatal risk factors for morbidity and growth in a birth cohort in Southern India. British Medical Journal Open 2014; 4: e005404. 10.1136/bmjopen-2014-005404 PMC4120427 25056979

[3] O’ Donovan SM, Cohort profile: the cork baseline birth cohort study: babies after scope: evaluating the longitudinal impact on neurological and nutritional endpoints. International Journal of Epidemiology 2015; 44: 764–775. 25102856 10.1093/ije/dyu157

[4] 1000 Days Partnership. 1,000 Days Partnership Progress Report 2013, 2013, 10000 Days, Washington. http://thousanddays.org/wp-content/uploads/2013/06/1000-Days-Brochure-Web.pdf

[5] Grantham-Mcgregor SM, Walker SP, Chang S. Nutritional deficiencies and later behavioural development. Proceedings of the Nutrition Society 2000; 59: 47–54. 10828173 10.1017/s0029665100000069

[6] Vickery J, Indigenous Insights into Oral History, Social Determinants and Decolonisation **In**: Anderson I, Baum F, Bentley M, eds. Beyond Bandaids: Exploring the Underlying Social Determinants of Aboriginal Health. Darwin: Co-operative Research Centre for Aboriginal Health, 2007, pp. 19–36.

[7] Heckman JJ. Schools, skills, and synapses In: Working Paper 14064. Cambridge, MA: National Bureau of Economic Research, 2008.

[8] Ou L, Ethnic and Indigenous access to early childhood healthcare services in Australia: Parents’ perceived unmet needs and related barriers. Australian and New Zealand Journal of Public Health 2010; 35: 30–37. 21299698 10.1111/j.1753-6405.2010.00633.x

[9] Government of Australia. Close the gap In: Indigenous Health Equality Summit. Canberra, 2008 http://www.health.nsw.gov.au/workforce/aboriginal/Documents/closing-the-gap-statement-intent.pdf

[10] Commonwealth of Australia. Closing the Gap Prime Ministers Report 2016. Canberra: Department of the Prime Minister and Cabinet, 2016.

[11] Council of Australian Governments (COAG). National Indigenous Reform Agreement (Closing the Gap). Canberra: Council of Australian Government, 2009.

[12] Family Matters. Family Matters – Kids Safe in Culture, Not in Care: An Invitation to Change the Lives of Aboriginal and Torres Strait Islander Children. Melbourne: SNAICC, 2014.

[13] Gray M, Hunter B, Lohoar S. Increasing Indigenous employment rates. In: Australian Institute of Health and Welfare, 2012 http://www.aihw.gov.au/uploadedFiles/ClosingTheGap/Content/Publications/2012/ctg-ip03.pdf

[14] Berry HL, Bowen K, Kjellstrom T. Climate change and mental health: a causal pathways framework. International Journal of Public Health 2010; 55: 123–132. 20033251 10.1007/s00038-009-0112-0

[15] Healing Foundation. Growing Our Children up Strong and Deadly – Healing for Children and Young People. 2013 http://healingfoundation.org.au/wordpress/wp-content/files_mf/1369185755GrowingourChildrenupsinglesfeb2013.pdf

[16] Parker R, Milroy H. Mental illness in Aboriginal and Torres strait Islander peoples In: Dudgeon P, Milroy H, Walker R, eds. *Working* Together: Aboriginal and Torres Strait Islander Mental Health and Wellbeing Principles and Practice. Australia: Telethon Kids Institute, 2014; 25–38.

[17] Australian Institute of Health and Welfare (Aihw). Aboriginal and Torres Strait Islander People with Disability: Wellbeing, Participation and Support. AIHW, Canberra, 2011.

[18] Gracey M, King M. Indigenous health part 1: determinants and disease patterns. Lancet 2009; 374: 65–75. 19577695 10.1016/S0140-6736(09)60914-4

[19] Taskforce 1000 Bulletin 3. Report on Government Services. ed. by Department of Human Services and Commission for Children and Young People Department of Human Services, 2015.

[20] Jackamos A. Taskforce 1000: a focus on vulnerable children across Victoria In: Arabena K, eds. Making the World of Difference: The First 1000 Days Scientific Symposium Report. Melbourne: The University of Melbourne, 2015, pp. 9–11.

[21] Mchugh A, Hornbuckle J. Maternal and child health model of care in the Aboriginal community controlled health sector. In: **Perth Aboriginal Health Council of Western Australia** Perth, WA: Aboriginal Health Council of Westerrn Australia, 2011 http://www.kemh.health.wa.gov.au/services/amssu/docs/AHCWA_Model_of_Care.pdf

[22] Comino E, Risk and protective factors for pregnancy outcomes for urban aboriginal and non-aboriginal mothers and infants: The Gudaga Cohort. Maternal and Child Health Journal 2012; 16: 569–578. 21505781 10.1007/s10995-011-0789-6

[23] Sainsbury H. Personal reflections of a project officer: working with Gudaga. Aboriginal and Islander Health Worker Journal 2009; 33: 4–5.

[24] Country WBT. Koorie Bubs Invited to Welcome Baby to Country Ceremony. 2015 (http://www.mildura.vic.gov.au/Latest-News/Koorie-bubs-invited-to-Welcome-Baby-to-Country-ceremony). Accessed 17 December 2015.

[25] Crook L, Waminda: mums and bubs program. Aboriginal and Islander Health Worker Journal 2012; 36: 17–19.

[26] Arabena K. The First 1000 Days: Catalysing equity outcomes for Aboriginal and Torres Strait Islander Children. Medical Journal of Australia 2014; 200: 442. 24794594 10.5694/mja14.00343

[27] National Children's Commisioner. Children's Rights Report 2015. Sydney: Australian Human Rights Commission, 2015.

[28] Bourke E, Bourke C. Aboriginal families in Australia In: Hartley R, eds. Families and Cultural Diversity in Australia. Canberra: Australian Institute of Family Studies, 1995; 48–69.

[29] Australian Health Ministers’ Advisory Council. Aboriginal and Torres Strait Islander Health Performance Framework 2014 Report. Canbera: AHMAC, 2015.

[30] Healing Foundation. Our Children, Our Dreaming: A Call for a More Just Approach for Aboriginal and Torres Strait Islander Children and Families. Secretariat of National Aboriginal and Islander Child Care (SNAICC), Australia, 2013, Australia.

[31] Save the Children. Statutory Child Protection and Care in South Australia: Submission to the Select Committee, 2014 https://www.savethechildren.org.au/__data/assets/pdf_file/0019/60454/Submission-to-the-on-Statutory-Child-Protection-and-Care-in-South-Australia.pdf

[32] Australian Red Cross. Aboriginal and Torres Strait Islander Strategy 2009–2015, 2009 http://www.redcross.org.au/files/ATSI_Strategy_2009.pdf

[33] World Vision Australia. Australian Programtic Strategic Plan. World Vision Australia (WVA), Australia, 2009.

[34] Bowes J, and Grace R. Review of Early Childhood Parenting, Education and Health Intervention Programs for Indigenous Children and Families in Australia. Closing the Gap Clearinghouse, AIHW, Canberra & Australian Institute of Family Studies, Canberra, 2014.

[35] Steering Committee for the Review of Government Service Provision (Scrgsp). Overcoming Indigenous Disadvantage: Key Indicators 2014. Canberra: Productivity Commission, 2014.

[36] Australian Department of Families, H., Community Services and Indigenous Affairs (Fahcsia). Footprints in Time: The Longitudinal Study of Indigenous Children - Key Summary Report. Canberra: Australian Department of Families, Housing, Community Services and Indigenous Affairs, 2012.

[37] Zhao Y, Decomposing indigenous life expectancy gap by risk factors: a life table analysis. Population Health Metrics 2013; 11: 1–9. 23360645 10.1186/1478-7954-11-1 PMC3585166

[38] Commonwealth of Australia. National Aboriginal and Torres Strait Islander Health Plan 2013–2023. Canberra: Australian Government, 2013.

[39] Walter M, Andersen C. Indigenous Statistics: A Quantitative Research Methodology. California: Left Coast Press, Inc., 2013.

[40] Wingard B, Johnson C, Drahm-Butler T. Aboriginal Narrative Practice: Honouring Storylines of Pride, Strength and Creativity. South Australia: Narrative Therapy, 2015.

[41] Kendall E, ‘Beyond the Rhetoric of participatory research in indigenous communities: advances in Australia over the last decade. Qualitative Health Research 2011; 21: 1719–1728. 21844284 10.1177/1049732311418124

[42] Cargo M, Mercer SL. The value and challenges of participatory research: strengthening its practice. Annual Review of Public Health 2008; 29: 325–350. 10.1146/annurev.publhealth.29.091307.083824 18173388

[43] Rigney LI. Internationalization of an Indigenous Anticolonial cultural critique of research methodologies: a guide to Indigenist research methodology and its principles. Wicazo Sa Review 1997; 14: 109–121.

[44] Kania J, Kramer M. Collective impact. Stanford Social Innovation Review Stanford University, Standford, California, 2011; 36–41.

[45] Arabena K, Making the World of Difference: The First 1000 Days Scientific Symposium Report. Melbourne: Onemda VicHealth Group, The University of Melbourne, 2015.

[46] Arabena K, Panozzo S, Ritte R. The First 1000 Days Researchers’ Forum Report. Melbourne: Onemda VicHealth Group, The University of Melbourne, 2015.

[47] Arabena K, Strengthening Families through the First 1000 Days Community Governance Symposium: Report. Melbourne: Onemda VicHealth Group, The University of Melbourne, 2015.

[48] Arabena K, Panozzo S, Ritte R. The First 1000 Days Policy and Implementers Symposium Report. Melbourne: Onemda VicHealth Group, The University of Melbourne, 2015.

[49] Ghera MM, The effects of foster care intervention on socially deprived institutionalized children's attention and positive affect: results from the BEIP study. Journal of Child Psychology and Psychiatry 2009; 50: 246–253. 19309327 10.1111/j.1469-7610.2008.01954.x

[50] Radford K, The Koori growing old well study: investigating aging and dementia in Urban Aboriginal Australians. International Psychogeriatrics 2014; 26: 1033–1043. 24507414 10.1017/S1041610213002561

[51] Campbell FA, Adult outcomes as a function of an early childhood educational program: an Abecedarian project follow-up. Developmental Psychology 2012; 48: 1033–1043. 22250997 10.1037/a0026644 PMC3989926

[52] Boffa J, Reducing the Harm from Alcohol, Tobacco and Obesity in Indigenous Communities: Key Approaches and Actions. Canberra: National Preventative Health Taskforce, 2009.

[53] Azzopardi PS, The quality of health research for young Indigenous Australians: systematic review. Medical Journal of Australia 2013; 199: 57–63. 23829266 10.5694/mja12.11141

[54] Center on the Developing Child at Harvard University (Cdchu). ‘Building the Brain's ‘Air Traffic Control’ System: How Early Experiences Shape the Development of Executive Function, Working Paper 11’, CDCHU, Cambridge, MA, 2011.

[55] Taylor KP, Thompson SC. Closing the (Service) gap: exploring partnerships between Aboriginal and Mainstream Health services. Australian Health Review 2011; 35: 297–308. 21871191 10.1071/AH10936

[56] Arabena K, Panozzo S, Ritte R. The First 1000 Days Policy and Implementers’ Symposium Report. Melbourne: Onemda VicHealth Group, The University of Melbourne, 2016.

[57] World Health Organisation (Who). Declaration of Alma Ata. International Conference on Primary Health Care. Alma-Ata, USSR, Geneva, 1978.

[58] Naccho. Schedule 5: core functions of primary health care in Aboriginal Community Controlled Health Services (Accchs) In: Constitution for the National Aboriginal Community Controlled Health Organisation. Canberra: National Aboriginal Community Controlled Health Organisation, 2011, pp. 54–58.

[59] Napier AD, Culture and health. Lancet 2014; 384: 1607–1639. 25443490 10.1016/S0140-6736(14)61603-2

[60] Fredericks B, Legge D. Revitalising Health for All: International Indigenous Representative Group. Learning from the Experience of Comprehensive Primary Health Care in Aboriginal Australia - a Commentary on Three Projects. Melbourne: The Lowitja Institute, 2011.

[61] Yap M, Biddle N. Indigenous fertility and family formation **In**: Centre for Aboriginal Economic Policy Research, ed. 2011 Census Papers. Canberra: Research School of Social Sciences, Australian National University, 2011.

[62] Mccalman J, Empowering families by engaging and relating Murri way: a grounded theory study of the implementation of the Cape York baby basket program. BMC Pregnancy Childbirth 2015; 15: 1–20. 25994123 10.1186/s12884-015-0543-y PMC4479238

[63] Mein J, Apunipima baby baskets. Obstetrics and Gynaecology Magazine 2011; 13: 21–23.

[64] Australian Institute of Aboriginal and Torres Strait Islander Studies (Aiatsis). Guidelines for Ethical Research in Australian Indigenous Studies’ (http://aiatsis.gov.au/research/ethical-research/guidelines-ethical-research-australian-indigenous-studies/rights-respect-and-recognition), 2016.

[65] National Health and Medical Council (Nhmrc). Values and Ethics, Guidelines for Ethical Conduct in Aboriginal and Torres Strait Islander Health Research. National Health and Medical Research Council (NHMRC), Commonwealth of Australia, Canberra, 2003.

[66] Johnston L, A review of programs that targeted environmental determinants of aboriginal and Torres Strait Islander health. International Journal of Environmental Research and Public Health 2013; 10: 3518–3542. 23939388 10.3390/ijerph10083518 PMC3774452

[67] Rowley K, Strengths and limitations of a tool for monitoring and evaluating first peoples’ health promotion from an ecological perspective. BMC Public Health 2015; 15: 1215. 26646295 10.1186/s12889-015-2550-3 PMC4672567

[68] Arabena K, Rowley K, Maclean S. Editorial issue 4 2014: building evidence about effective health promotion in aboriginal and Torres Strait islander communities. Australian Journal of Primary Health 2014; 20: 317–318. 25354980 10.1071/PYv20n4_ED

[69] Wiseman J, Nichols D. Finding common ground [Community Development Projects Can Make a Difference.]. Eureka Street 2003; 13: 8–11.

[70] Sherwood J, Indigenous management model. Aboriginal & Islander Health Worker Journal 1999; 23: 16–19.

[71] De Crespigny Charlotte, A ‘Partnership model’ for ethical Indigenous research. Collegian 2004; 11: 7–13.

[72] Department of Health and Ageing. National Aboriginal and Torres Strait Islander Health Plan 2013–2023 In: Closing the Gap, Australian Government, Canberra.2013 URL: http://www.health.gov.au/internet/main/publishing.nsf/content/B92E980680486C3BCA257BF0001BAF01/$File/health-plan.pdf

[73] United Nations. United Nations Declaration on the Rights of Indigenous Peoples, United Nations, New York, 2008 http://www.un.org/esa/socdev/unpfii/documents/DRIPS_en.pdf

[74] Prior D. Decolonising research: a shift toward reconciliation. Nursing Inquiry 2007; 14: 162–168. 17518828 10.1111/j.1440-1800.2007.00361.x

[75] Nakata M. The cultural interface. Australian Journal of Indigenous Education 2007; 36: 7–14.

[76] Brown VA, Lambert JA. Collective Learning for Transformational Change: A Guide to Collaborative Action. UK: Routledge, 2013.

[77] Lexa FJ. Principled leadership. Journal of American College of Radiology 2010; 7: 529–5230. 10.1016/j.jacr.2010.03.022 20630389

[78] Hunter B. Benchmarking the Indigenous sub-sample of the longitudinal study of Australian children. Australian Social Policy 2008; 7: 61–84.

[79] Freemantle J, Indigenous mortality (Revealed): the invisible illuminated. American Journal of Public Health 2015; 105: 644–652. 25211754 10.2105/AJPH.2014.301994 PMC4358192

[80] Freemantle J, Victorian Childhood Mortality Study: Patterns, Trends and Disparities in Mortality between Aboriginal and Non-Aboriginal Infants and Children, 1999–2008. Melbourne: The Lowitja Institute, 2014.

[81] National Assessment Program Literacy and Numeracy (Naplan). Achievement in Reading, Persuasive Writing, Language Conventions and Numeracy - National Report for 2015. Sydney: Australian Curriculum, Assessment and Reporting Authority (ACARA) 2015, 2015.

[82] Anderson I, Indigenous and Tribal Peoples’ Health (the Lancet-Lowitja Institute Global Collaboration): a population study. Lancet 2016. [Epub ahead of print]. 10.1016/S0140-6736(16)00345-7 27108232

[83] Snodgrass JJ. Health of Indigenous circumpolar populations. Annual Review of Anthropology 2013; 42: 69–87.

[84] Obimbo EM. Primary health care, selective or comprehensive, which way to go? East African Medical Journal 2003; 80: 7–10. 12755235 10.4314/eamj.v80i1.8659

